# Coronary Artery Ectasia: A Case Report Discussing the Causes, Diagnosis, and Treatment

**DOI:** 10.7759/cureus.14431

**Published:** 2021-04-12

**Authors:** Rana Al-Zakhari, Safa Aljammali, Sean Galligan, Francesco Rotatori

**Affiliations:** 1 Internal Medicine, Richmond University Medical Center, Staten Island, USA; 2 Cardiology, Richmond University Medical Center, Staten Island, USA; 3 Interventional Cardiology, Richmond University Medical Center, Staten Island, USA

**Keywords:** coronary artery ectasia, coronary angiography, coronary computed tomography angiogram, coronary magnetic resonance angiogram

## Abstract

The localized or diffused dilation of a coronary artery lumen is referred to as coronary artery ectasia (CAE). Though it is well recognized, CAE is a rare finding that is encountered in the diagnostic procedure of coronary angiography. This form of atherosclerotic coronary artery disease (CAD) can be found in 1.4-4.9% of all coronary angiography patients. CAE can manifest in combination with stenotic lesions or present as an isolated condition. Its risk factors are similar to those of atherosclerosis. The underlying pathophysiology involves a vascular remodeling response to atherosclerosis. Enzymatic degradation by matrix metalloproteinases (MMP) and accumulation of lipoproteins play an important role in the remodeling process. CAE can be diagnosed with the help of imaging modalities such as coronary CT angiogram (CTA) and coronary magnetic resonance angiogram (MRA); coronary angiography is considered the gold standard procedure. The management strategies include treating the cardiovascular risk factors, prevention of thromboembolic events, and percutaneous/vascular revascularization. CAE can be managed medically, but percutaneous/surgical revascularization [coronary artery bypass grafting (CABG)] is an option to treat patients with co-existing symptomatic obstructive lesion refractory to medical treatment. Further trials are required to optimize the management guidelines related to CAE. In this report, we describe the case of a 42-year-old man with a past medical history of hypertension, hyperlipidemia, and asthma who presented with shortness of breath and minimally elevated troponin level. Coronary angiography revealed three vessels with ectasia and severe left ventricular dysfunction on ventriculography.

## Introduction

Historically, the aneurysmal dilation of coronary arteries has been described using the following two terms: coronary artery ectasia (CAE) and coronary artery aneurysm [1]. A coronary segment with focal dilation greater than or equal to 1.5 times the adjacent normal segment is described by the term coronary artery aneurysm [2]. On the other hand, diffused aneurismal lesions are referred to as CAE. The lack of data to support a specific treatment plan combined with variable presentations and poor understanding of coronary ectasia mechanisms makes it difficult to manage coronary ectasia patients. In this report, we focus on the definitions, pathophysiology, epidemiology, clinical presentations, risk factors, and ultimately the management methods related to aneurysmal coronary artery disease (CAD).

## Case presentation

The patient was a 42-year-old African American male experiencing acute shortness of breath who had been brought to the emergency room by the emergency medical services (EMS). His past medical history included hyperlipidemia, hypertension, asthma, and the use of tobacco and marijuana. For the past two weeks, he had been experiencing dyspnea caused by exertion, which had become aggravated in the immediate past week. The patient did not have palpitations or chest pain. His blood pressure was noted to be 144/90 mmHg. Electrocardiogram (ECG) showed sinus rhythm with left ventricular hypertrophy with non-specific ST-T abnormalities. His troponin levels were minimally elevated at 0.2 ng/L (normal level: <0.04 ng/L). An echocardiogram showed moderate-to-severe left ventricular dysfunction [visually estimated ejection fraction (EF) was 35-40%] with global hypokinesia, mild mitral, tricuspid, aortic, and pulmonary regurgitation, as well as moderate left atrial dilatation. Pro-brain natriuretic peptide (proBNP) level was 1,476 pg/mL (normal level: 300 pg/mL); liver function tests and creatinine were found to be mildly elevated. On the drug screen, the patient tested positive for marijuana and cocaine. The patient was given furosemide and aspirin immediately.

The patient was admitted with a working diagnosis of possible non-ST segment elevation myocardial infarction (NSTEMI) versus hypertensive emergency. One day later, a nuclear stress test was done, which showed that the ventricular apex, as well as the lateral and inferior walls of the left ventricle, had large fixed perfusion defects. The inferior and apex walls also had defects represented by a small area of stress-induced ischemia superimposed on a large chronic infarct. Severely reduced EF was observed: 21% at rest and 16% when under stress.

**Figure 1:**
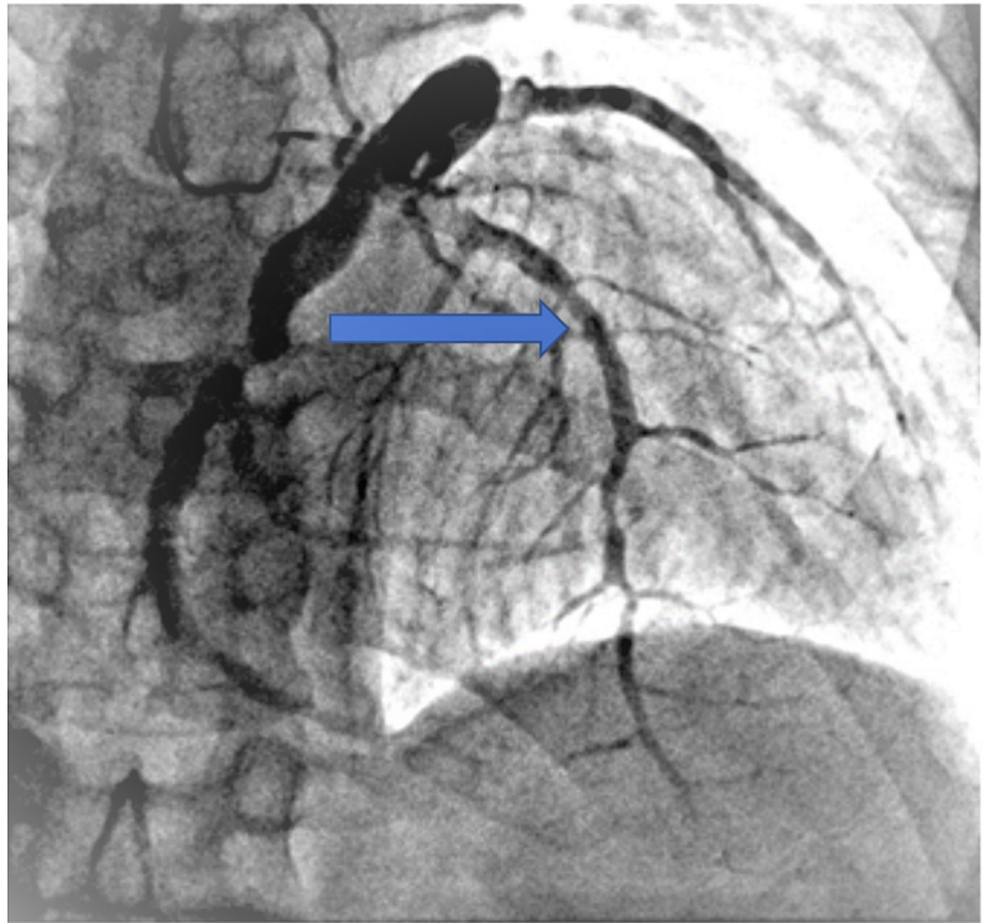
Left anterior descending artery was found to be aneurysmal and ectatic with sequential discrete lesions and 80% stenosis of the middle segment and 100% occlusion of the distal segment (blue arrow)

**Figure 2:**
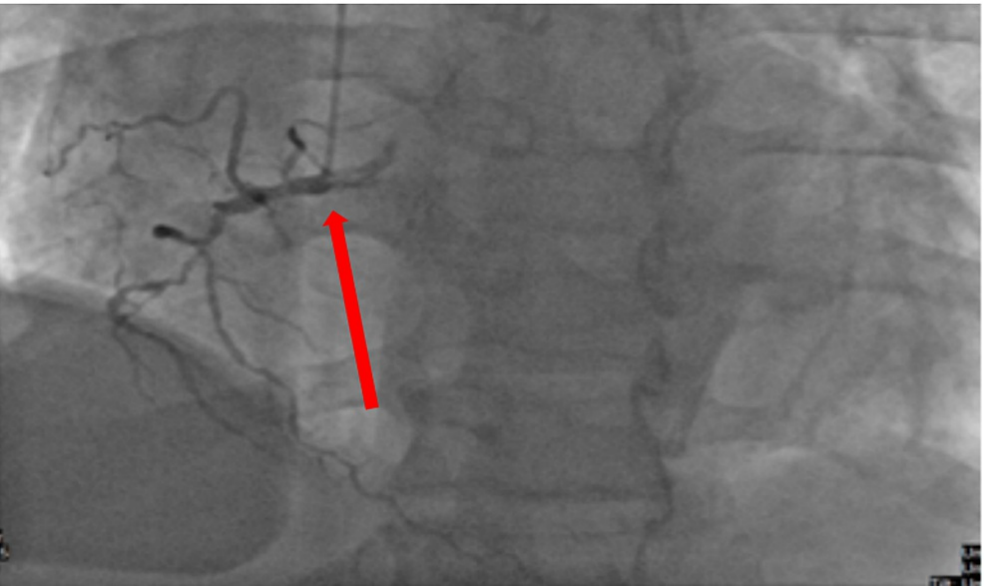
Right coronary artery showed proximal chronic total occlusion (red arrow)

**Figure 3:**
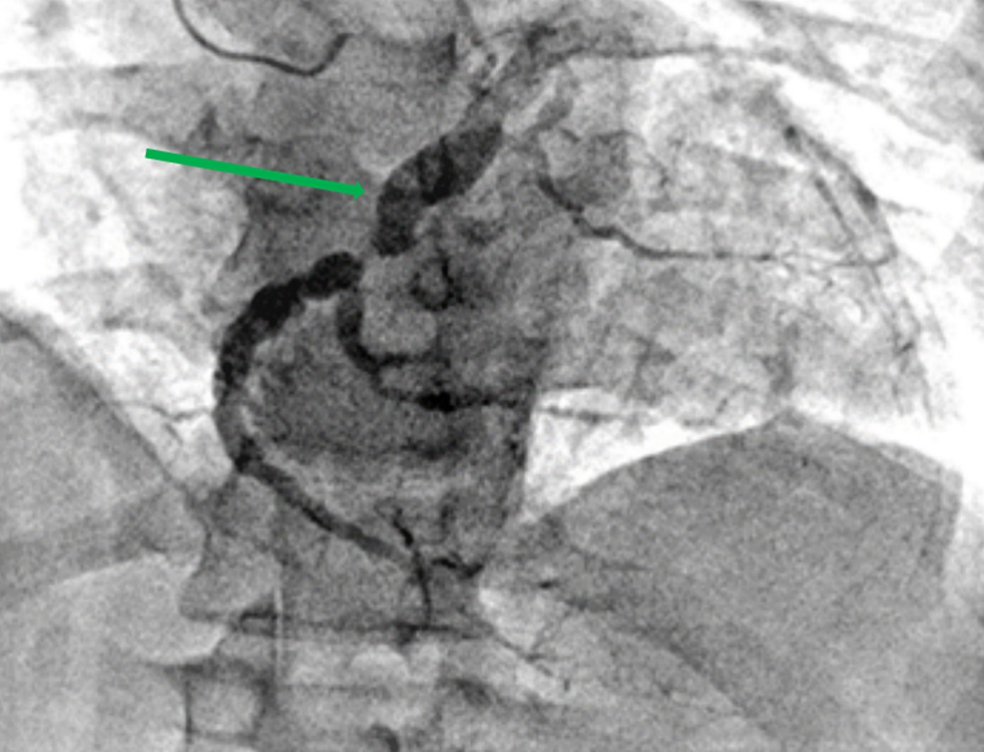
Left circumflex was also found to be a large caliber ectatic vessel with slow flow and critical 90% stenosis right before bifurcating in the left posterior descending artery and obtuse marginal 2 (green arrow)

Cardiac catheterization of the patient revealed severe dysfunction in the left ventricular and a severe diastolic dysfunction given high left ventricular end-diastolic pressure (LVEDP). Coronary angiography showed that the left anterior descending artery (LAD) was aneurysmal and ectatic combined with 80% middle segment tandem stenosis and 100% distal segment occlusion following discrete lesions; left circumflex (LCX) was found to be a large caliber ectatic vessel with slow flow and critical 90% stenosis right before bifurcating in the left posterior descending artery (PDA) and obtuse marginal 2 (OM2). The right coronary artery (RCA) was found to be an ectatic vessel in the proximal portion having 100% chronic total occlusion (CTO) (Figures 1, 2, 3).

The patient was referred to a specialized cardiac center for further intervention. Cardiac catheterization was done; two drug-eluting stents (zotarolimus), one measuring 5.0 x 15 mm followed by another one of 5.0 x 22 mm, were placed in distal LCX. Post-dilation was performed with a non-compliant (NC) balloon of 6.0 x 12 mm at 16 atm. The procedure was done with hemodynamic support of Impella LP 2.5 (Abiomed, Danvers, MA). For the LAD artery, staged percutaneous coronary intervention (PCI) was scheduled, but the patient was lost to follow-up.

A few months later, his left ventricular EF improved to 40% and the patient did not require an implantable cardioverter-defibrillator (ICD).

**Figure 4:**
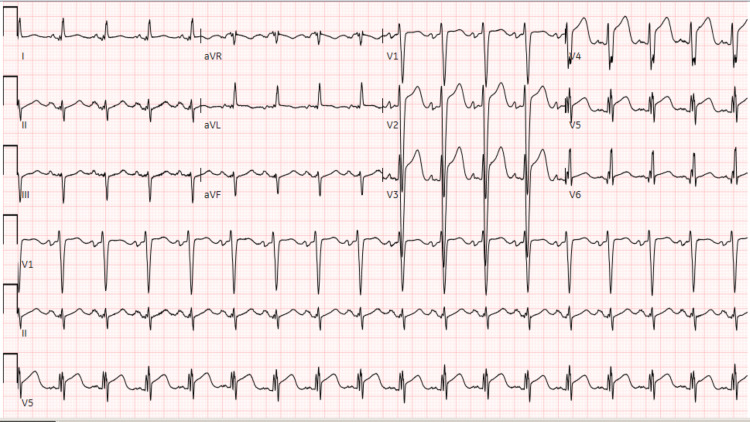
ECG shows ST elevation in the anterolateral leads concerning for acute MI/STEMI ECG: electrocardiogram; MI: myocardial infarction; STEMI: ST-elevation myocardial infarction

**Figure 5:**
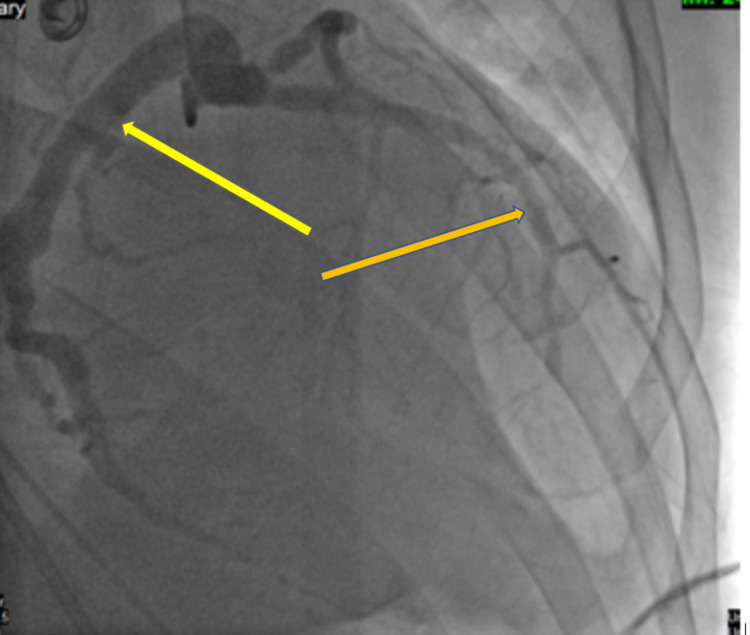
After percutaneous angioplasty, a stent was placed to the left anterior descending artery (orange arrow), patent circumflex artery, and moderate diffuse ectasia of the left anterior descending artery (yellow arrow)

However, one year later, the patient returned to the emergency room complaining of acute shortness of breath. He was in respiratory distress that required ventilatory support. After the intubation, the patient went into cardiac arrest secondary to pulseless electrical activity (PEA); return of spontaneous circulation (ROSC) was achieved in five minutes. Troponin level was found to be elevated at 0.9 ng/L. The ECG showed ST elevation in the anterolateral leads (Figure 4). Cardiac catheterization was done emergently, and angiography showed 100% CTO of the RCA, a patent stent in the LCX, and a moderate diffuse ectasia of LAD with 100% thrombotic occlusion of the distal LAD (Figure 5). A drug-eluting stent of 3.0 x 28 mm was placed. Four days later, the patient was extubated; his condition improved, and he was discharged home.

## Discussion

The term CAE refers to an aneurysmal dilation of the coronary artery associated with a disturbed coronary blood flow. Based on the Coronary Artery Surgery Study (CASS) registry, the definition of CAE includes dilation of coronary artery involving a diameter greater than the adjacent normal coronary artery by 1.5 times [3]. A 3:1 male-to-female ratio with a prevalence rate of 1.2-4.9% has been reported [4].

Based on the morphology and the extent of the coronary arteries’ involvement, we can divide CAE into different groups [1].

Classification based on morphology includes the following types:

1. Saccular: the transverse diameter is more than the longitudinal dimension.

2. Fusiform: the transverse diameter is lower than the longitudinal dimension.

Classification based on the extent includes the following types:

1. Type 1: diffuse ectasia involving aneurysmal lesions in two vessels

2. Type 2: discrete ectasia in one vessel with diffuse ectasia in another vessel

3. Type 3: one vessel having diffuse ectasia

4. Type 4: one vessel having discrete ectasia

In a single observational study involving approximately 5,000 patients, CAE was reported mostly in proximal-mid-RCA (68%), followed by proximal LAD in 60% of cases, and LCX in 50% of the cases. Left main coronary artery ectasia (LMCA) is an extremely rare occurrence, which accounts for 0.1% of cases [3,5].

In one study involving both children and adults, the most common cause was found to be the Kawasaki disease (widespread inflammation of primarily medium-sized muscular arteries) [6]. In another study, more than 50% of adult cases were due to atherosclerosis [1].

The mechanism of ectasia in atherosclerosis is considered to be a consequence of vessel lumen dilatation caused by expansive vascular remodeling to atherosclerotic growth plaques. There is also an expansion of the media and the external elastic membrane resulting in the enlargement of the internal diameter of the vessel. Other causes responsible for the remodeling process include tunica media thinning related to severe chronic inflammation and enzymatic degradation of lytic enzymes like extracellular matrix-by-matrix metalloproteinases (MMP), accumulation of lipoproteins into the intima, inflammatory cell infiltration, renin-angiotensin system activation, and generation of oxidative stress that leads to excessive expansive arterial remodeling and ectasia formation [7].

CAE can also be caused by apical hypertrophic cardiomyopathy (HCM) secondary to high tension wall. Post-percutaneous coronary interventions such as stent placement, atherectomy, and balloon angioplasty also exhibit aneurysm formation as a consequence of iatrogenic injury to the blood vessel media [8].

In general, males are more prone to CAE than females, and the biggest risk factor is hypertension [3]. A low incidence of CAE has been found among diabetic patients. This could be attributed to negative remodeling along with the down-regulation of MMP in response to atherosclerosis [9]. The habit of smoking has been associated more with patients suffering from CAE than patients with CAD. Regardless of smoking, another independent predictor of CAE is cocaine use [10].

Coronary angiography has been considered the gold standard procedure to diagnose CAE. The disturbances in washout and blood flow filling related to the severity of ectasia can be shown with the help of angiography. The local dye deposition in the dilated coronary segment and the segmental backflow phenomenon known as delayed antegrade dye filling are the signs of stagnant and turbulent flow signs indicated in angiography [11,12]. Nowadays, CAE can be also diagnosed with the help of other modalities such as coronary CT angiogram (CTA) and coronary magnetic resonance angiogram (MRA) [8].

A false aneurysm is defined as a discrete enlargement of the lumen of a coronary artery after the rupture of large lipidic plaque. Differentiating between true and false aneurysms due to plaque rupture as well as evaluation of pathologies and luminal characteristics is important. In such cases, intravascular ultrasound (IVUS) and optical coherence tomography (OCT) play a vital role [13].

Apart from some exceptions, managing the disease is similar to how CAD is managed. Stent angioplasty and surgical interventions are also employed. Medical therapy for CAE has not received any attention from random studies so far. Because of the co-existence of CAE with obstructive coronary lesions in the great majority of patients, all patients are recommended to use aspirin [14].

There have not been any prospective random studies evaluating the role of adenosine diphosphate inhibitors as part of therapy. It is still controversial to use calcium channel blockers for angina and vasodilator effects like nitrates for therapy. Since it is possible that thromboembolic events may be triggered by the significant flow stagnation in the ectatic segments [15], it was strongly suggested to use warfarin as the basic treatment for achieving long-term anticoagulation in one study [16]. Of course, these patients may benefit from using lipid-lowering agents since one of the risk factors is dyslipidemia [17]. The progression of the disease can be reduced if the related cardiovascular risk factors like hypertension and diabetes are controlled.

Patients having concurrent obstructive lesions along with myocardial ischemia symptoms who are not responding to medical treatment can be chosen for a procedure involving percutaneous transluminal coronary angioplasty (PTCA) and surgical coronary artery vascularization with coronary artery bypass grafting (CABG) [18].

PCI is challenging due to the necessity of using large-size devices and, sometimes, off-label stents for peripheral arteries. Higher incidence of no-reflow and distal embolization, and higher rates of subsequent stent thrombosis, repeat revascularization, and long-term mortality have also been associated with PCI. Another major challenge during PCI is the thrombus burden within the aneurysm, which raises the potential need for a covered stent [19].

## Conclusions

CAE is a variant of CAD and atherosclerosis, and it is less commonly a consequence of the autoimmune vasculitis phenomenon. In order to understand the natural history, pathophysiology, phenotypic features, and optimal treatment methods related to this condition more comprehensively, we need to carry out further investigations in symptomatic and non-symptomatic patients. Although no guidelines are present at the moment regarding the treatment of CAE, the process of managing CAE is similar to that of CAD. This lack of evidence impedes the management of CAE and highlights the necessity of further detailed studies to gain more understanding about how to manage this disease.
